# Grass-Shrub Associations over a Precipitation Gradient and Their Implications for Restoration in the Great Basin, USA

**DOI:** 10.1371/journal.pone.0143170

**Published:** 2015-12-01

**Authors:** Maike F. Holthuijzen, Kari E. Veblen

**Affiliations:** Dept. of Wildland Resources and Ecology Center, Utah State University, Logan, Utah, United States of America; Estacion Experimental de Zonas Aridas - CSIC, SPAIN

## Abstract

As environmental stress increases positive (facilitative) plant interactions often predominate. Plant-plant associations (or lack thereof) can indicate whether certain plant species favor particular types of microsites (e.g., shrub canopies or plant-free interspaces) and can provide valuable insights into whether “nurse plants” will contribute to seeding or planting success during ecological restoration. It can be difficult, however, to anticipate how relationships between nurse plants and plants used for restoration may change over large-ranging, regional stress gradients. We investigated associations between the shrub, Wyoming big sagebrush (*Artemisia tridentata* ssp. *wyomingensis*), and three common native grasses (*Poa secunda*, *Elymus elymoides*, and *Pseudoroegneria spicata)*, representing short-, medium-, and deep-rooted growth forms, respectively, across an annual rainfall gradient (220–350 mm) in the Great Basin, USA. We hypothesized that positive shrub-grass relationships would become more frequent at lower rainfall levels, as indicated by greater cover of grasses in shrub canopies than vegetation-free interspaces. We sampled aerial cover, density, height, basal width, grazing status, and reproductive status of perennial grasses in canopies and interspaces of 25–33 sagebrush individuals at 32 sites along a rainfall gradient. We found that aerial cover of the shallow rooted grass, *P*. *secunda*, was higher in sagebrush canopy than interspace microsites at lower levels of rainfall. Cover and density of the medium-rooted grass, *E*. *elymoides* were higher in sagebrush canopies than interspaces at all but the highest rainfall levels. Neither annual rainfall nor sagebrush canopy microsite significantly affected *P*. *spicata* cover. *E*. *elymoides* and *P*. *spicata* plants were taller, narrower, and less likely to be grazed in shrub canopy microsites than interspaces. Our results suggest that exploring sagebrush canopy microsites for restoration of native perennial grasses might improve plant establishment, growth, or survival (or some combination thereof), particularly in drier areas. We suggest that land managers consider the nurse plant approach as a way to increase perennial grass abundance in the Great Basin. Controlled experimentation will provide further insights into the life stage-specific effectiveness and practicality of a nurse plant approach for ecological restoration in this region.

## Introduction

Plant spatial distributions reflect biological processes and are studied to gain insight into plant-plant interactions [1–3]. Aggregated (as compared to random or hyper-dispersed) plant distributions can indicate positive or “facilitative” relationships [3, 4], whereby one plant (a beneficiary) gains an advantage by growing in close proximity to its neighbor (nurse or benefactor). In facilitative relationships, the neighbor often provides a favorable microclimate [5–8] or defense against large mammal herbivory [9, 10]. The frequency and importance of facilitative plant interactions are predicted to increase with increasing environmental stress [11] (but see [12, 13]), particularly in arid and semi-arid ecosystems [4, 14].

Better understanding of nurse-beneficiary plant relationships may improve establishment of native plants in ecological restoration settings [15–17], particularly when nurse plants can ameliorate extreme abiotic stress imposed by drought and extreme temperatures [15, 16, 18]. Nurse plant sub-canopies create microsites with favorable abiotic conditions characterized by improved soil moisture [6, 18], moderated soil temperature [8], increased soil nutrient availability [19] and decreased solar radiation [18, 20]. Nurse plants also may protect sub-canopy plants from biotic stresses such as ungulate grazing [9, 21], a prevalent stress in arid and semi-arid ecosystems. Conversely, potential nurse plants may not always be beneficial in restoration settings. For example, microsites provided by sub-canopies of potential nurse plants may be less hospitable to target plants if they increase abundance or biomass, and therefore competitive effects, of other herbaceous species [22] particularly invasive species [23]. Similarly, nurse plants can themselves compete with sub-canopy species for resources such as light, nutrients or water, especially in extremely stressful environments [24].

In some cases, native species establishment or growth may require facilitation by other plants [25]. A growing number of studies have specifically considered use of nurse plants to improve establishment of native species in ecological restorations. Examples include use of tussock grasses to improve native shrub establishment [18] and shrubs to improve herbaceous seedling survival [26] and tree seedling establishment [15]. These studies provide evidence that using existing vegetation (e.g., shrubs) as nurse plants can be a viable tool for maximizing establishment and survival of restoration plantings [16].

**Fig 1:**
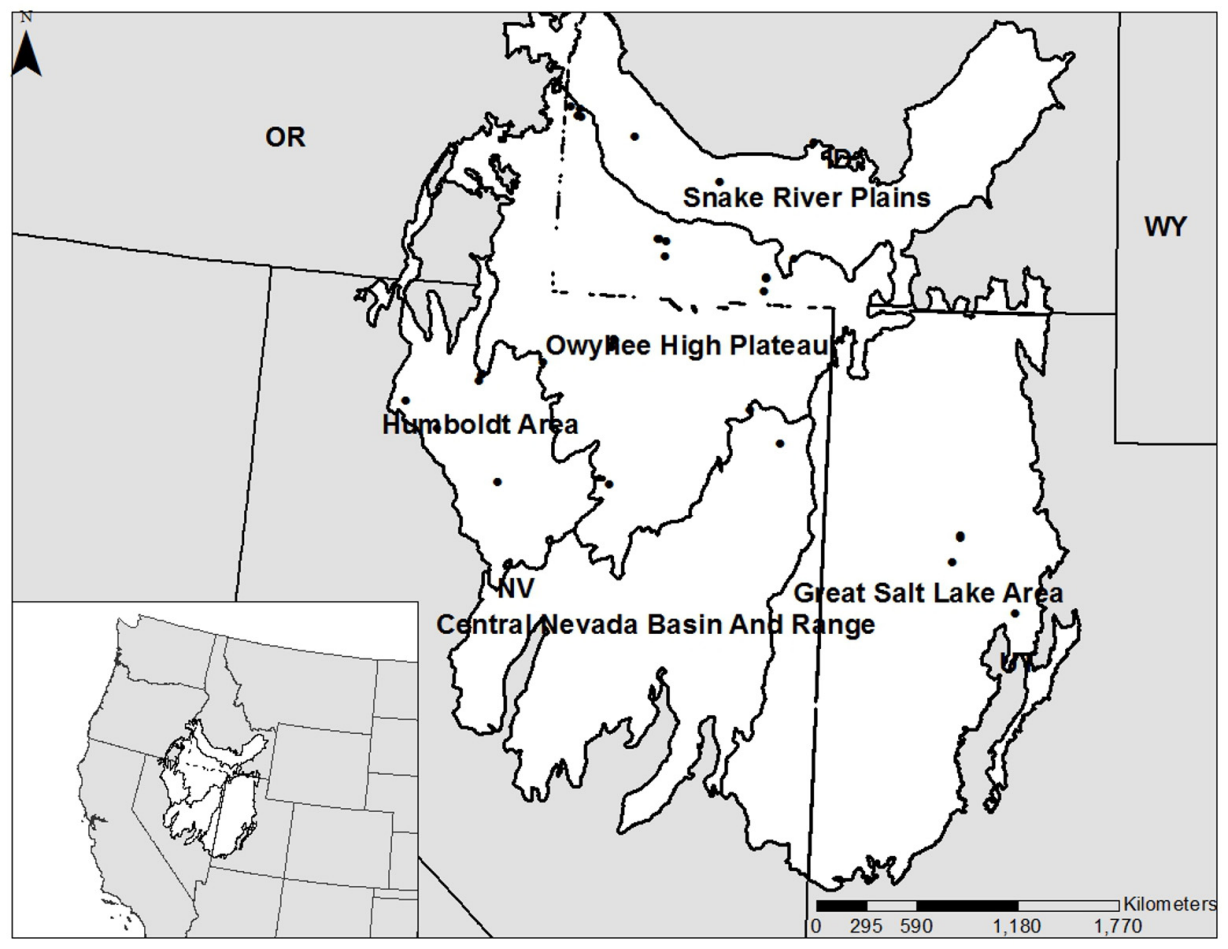
Sampling sites in UT, ID, and NV. Sites were located in five major land resource areas (geographically associated land units) across the Great Basin, USA (inset). Maps were created in ArcMap v10.1. Major land resource area spatial data was obtained from the Natural Resources Conservation Service’s Geospatial Data Gateway (https://gdg.sc.egov.usda.gov/).

Because plant-plant interactions often shift from positive at high stress to negative at low stress [11, 27, 28], the efficacy of nurse plants as restoration tools likely also varies over regional gradients (that range from high to low stress). One region that would benefit from a better understanding of the utility of nurse plants is the Great Basin, USA (Fig 1), an area dominated by sagebrush steppe, one of the most endangered ecosystems of western North America [29]. Native plant restoration is a major priority in this region [30] because increasing the abundance of native perennial grasses can improve resistance to invasions by non-native species such as *Bromus tectorum* (cheatgrass) [31, 32]. *Artemisia tridentata* ssp. *wyomingensis* (Wyoming big sagebrush), the dominant shrub throughout much of this region at low to mid elevations, has been shown to facilitate common perennial grasses in this region [33]. It creates favorable sub-canopy microsites by reducing evapotranspiration [5], mediating soil temperatures [7], increasing soil water via hydraulic lift [34], and concentrating soil nutrients [35].

It is not clear, however, if or to what extent *A*. *tridentata* would be beneficial as a nurse plant for native perennial grasses throughout the Great Basin region (~32 million hectares) [30] that receives a wide range of annual rainfall (~200–450 mm) [36]. Field studies of grass-shrub relationships in the Great Basin have occurred at single sites [17] and in the northwestern portion of this ecosystem (Oregon, US) [7, 37], but only one [37] explicitly examined how these relationships might change over stress gradients. Targeting nurse shrub sub-canopies as planting or seeding microsites may improve restoration of perennial grasses in drier areas of the Great Basin where biotic or abiotic stress is high, but may be less beneficial in more moderate (e.g., wetter) areas. Alternatively, herbaceous plants could perform better in interspace microsites where there is less woody plant competition. Moreover, traits such as growth form or life history, may influence how different species respond to potential nurse shrubs [11, 38]. Other plant attributes, such as plant size and reproductive potential also may respond (positively or negatively) to microsite, which could have important implications for long-term restoration success.

**Fig 2:**
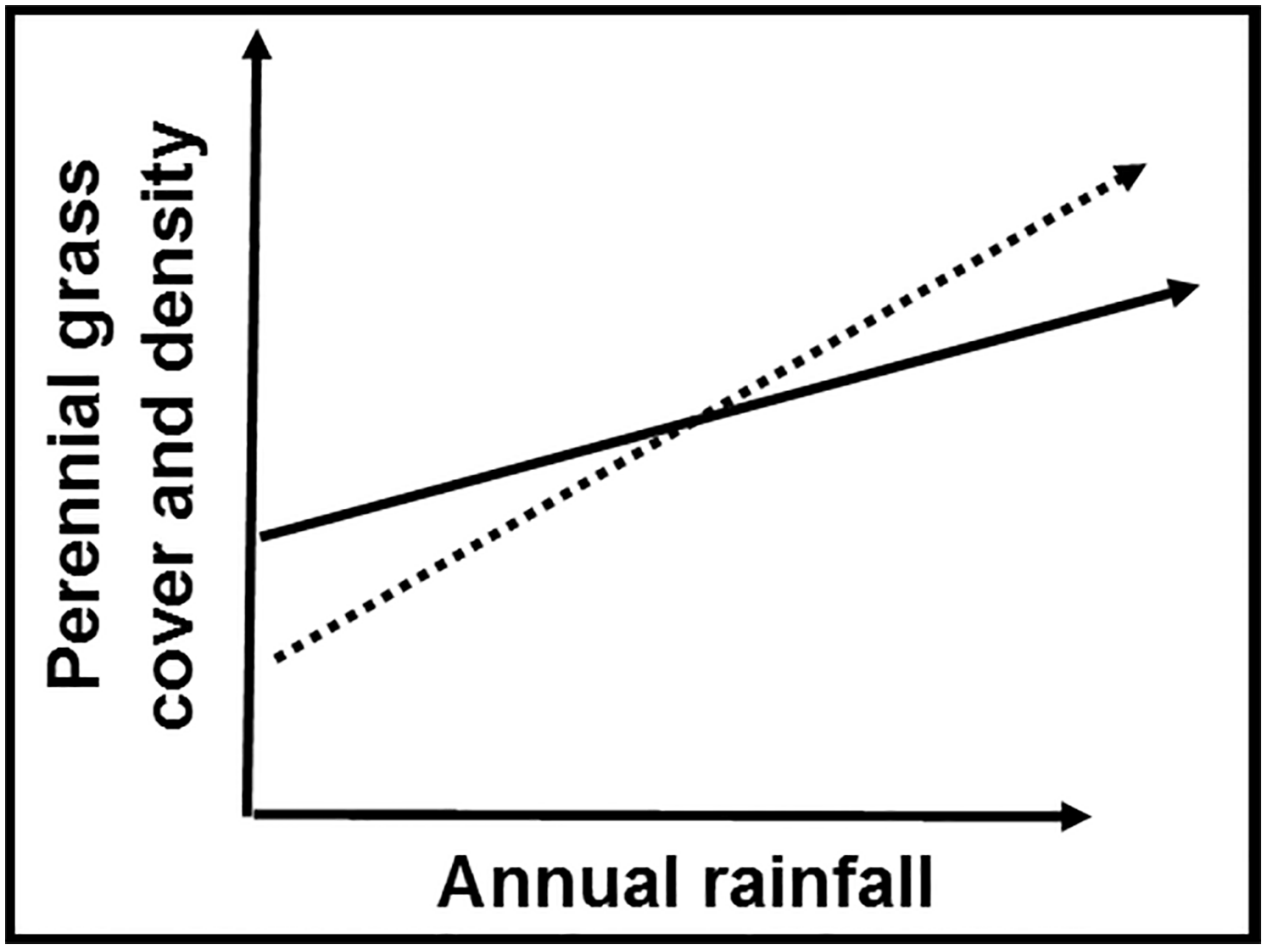
Hypotheses. At low rainfall levels, absolute cover and density of perennial grasses is greater in sub-canopy (solid line) than interspace (dashed line) microsites (i.e., positive grass-shrub relationship). As rainfall increases, the relationship reverses; at greater rainfall levels, absolute cover and density of perennial grasses are greater in interspace than sub-canopy microsites (i.e., negative grass-shrub relationship).

Understanding how a Great Basin-wide rainfall gradient affects associations between *A*. *tridentata* and perennial grasses could help restoration practitioners determine where, within the Great Basin, *A*. *tridentata* nurse plants have the highest potential to improve growth and establishment of native grasses. We focused on three cool-season perennial grass species that are of interest for use in restoration of Great Basin plant communities. In accordance with the stress gradient hypothesis [39], we hypothesized that, for all three grass species, the frequency of positive grass-shrub associations would increase as moisture stress increased (Fig 2). Specifically, we predicted that cover and density of grasses would be 1) greater in sub-canopy than interspace microsites at low rainfall, and 2) greater in interspace than sub-canopy microsites at high rainfall (Fig 2). We also predicted that densities and sizes of grasses rooted near the shrub canopy dripline (i.e., in “edge” microsites) would be intermediate between sub-canopies and interspaces at both high and low rainfall.

## Methods

### Study area and site selection

The study area encompassed *A*. *tridentata* ssp. *wyomingensis*-dominated areas across the Great Basin, USA (Fig 1). Our study sites were located within the five geographically associated land units, major land resource areas (MLRAs) [40], that contain plant communities dominated by *A*. *tridentata* ssp. *wyomingensis* [41] (Fig 1). We sampled over five MLRAs to capture the heterogeneity in climate and geology of the Great Basin region. Sampling occurred over three years (2012–2014) to obtain sufficient sample size for data analysis. Precipitation is variable throughout the region, though most of it falls November through May as snow and rain. The ~80-year average growing season (March-June) rainfall for weather stations located closest to our field sites (Boise, ID; Twin Falls, ID; Winnemucca, NV; Tuscarora, NV; and Tooele, UT) is 102.33mm. Yearly averages were 96.2 mm (2012), 74.2 mm (2013), and 33.8 mm (2014) [42].

We used a database of 826 candidate sites [43] to identify sites characterized by < 40% cover of *Bromus tectorum*. This species is an invasive, widespread plant of great economic and ecological concern in the Great Basin [44], and native plant restoration efforts may be most likely to succeed in less-invaded areas [45]. Other site criteria included presence of native perennial grasses and no recent (50 year) history of burning or land treatments. All sites had a history of spring and fall grazing in the five years prior to sampling. We used the Natural Resources Conservation Service Ecological Site Description (ESD) system [41] to select sites described as having loamy soils, a shrub overstory of *Artemisia tridentata spp*. *wyomingensis* (Wyoming big sagebrush), a dominant perennial grass understory of *Pseudoroegneria spicata* (bluebunch wheatgrass) or *Achnatherum thurberianum* (Thurber’s needlegrass), and a sub-dominant herbaceous understory of *Elymus elymoides* (squirreltail) and *Poa secunda* (Sandberg’s bluegrass). We retained 195 sites that met these criteria.

We determined mean rainfall (1981–2010) for each of the 195 candidate sites with a Parameter-Elevation Regressions on Independent Slopes Model (PRISM) one of the most widely used and accurate weather models available [36] for the study area. We prioritized sites with the greatest perennial grass cover and least cover of *Bromus tectorum* (cheatgrass). We then visited sites that were selected to represent a rainfall gradient in this region, as well as geographic variability across and within MLRAs. Final selection of our 32 field sites was based on field visits confirming minimal *B*. *tectorum* invasion and dominance by native perennial grasses (which were mostly *P*. *secunda* and *E*. *elymoides*, the grasses described as being sub-dominant for sites in their ESD reference state), as well as good accessibility (150–600 m from access roads) and evidence of minimal to moderate recent livestock grazing. All sites were located on public land managed by the Bureau of Land Management (BLM), and evidence of livestock grazing was based on field inspections and BLM grazing records. Sites sampled in 2012 (n = 20), 2013 (n = 9), and 2014 (n = 3) ranged in mean annual rainfall from 220 mm to 378 mm (see S1 Table for rainfall values at each site).

### Focal species

We selected three cool-season, native perennial grass species, *Poa secunda*, *Elymus elymoides*, and *Pseudoroegneria spicata* to represent caespitose bunchgrass growth forms most commonly seen in the Great Basin, namely, shallow-rooted tufted [46], medium-rooted [47], and deep-rooted, respectively [48]. *Pseudoroegneria spicata* can reach a rooting depth of 200 cm [48], while the maximum rooting depths of *E*. *elymoides* and *P*. *secunda* are 100 cm [47] and less than 100 cm [46], respectively. *Poa secunda* and *E*. *elymoides* were common across the entire range of rainfall whereas *P*. *spicata* was less common, especially in dry sites (S1 Table). All three species are common components of both native plant communities and restoration seed mixes in the Great Basin [49, 50]. *Artemisia tridentata ssp*. *wyomingensis* (Wyoming big sagebrush) is a perennial, evergreen shrub that can live over 100 years [51]. These shrubs often have rounded, asymmetrical crowns and typically range in height from 46–76 cm [52]. The root system includes a taproot that can reach depths of 150–240 cm and a shallow network of lateral roots reaching 90–150 cm from the shrub base [51].

### Sampling

We investigated how associations between Wyoming big sagebrush (*A*. *tridentata ssp*. *wyomingensis*) and three common native perennial grasses (*P*. *secunda*, *E*. *elymoides*, and *P*. *spicata*) responded to an annual rainfall gradient. In 2012–2014 field sampling occurred between May 15^th^ and July 30^th^ to capture peak herbaceous biomass. We sampled *P*. *secunda* at 29 sites, *E*. *elymoides* at 27 sites, and *P*. *spicata* at14 sites (S1 Table). At each of the 32 sites we sampled 25–33 sagebrush individuals; all (potentially co-occurring) target grass species beneath a given shrub were sampled. Our sampling criteria for sagebrush shrubs included: height > 40cm (at the tallest portion of live canopy), width > 40cm (at the longest axis of canopy and its perpendicular length), > 50% live canopy (visually estimated), and presence of a single clearly discernable base stem; approximately 80% of shrubs fit within this criteria. To select focal shrubs we walked along three 50-m lines that radiated, at equal spacing, from a single randomly selected point. Every five meters we selected the closest shrub, alternating between left and right sides of the line. If a shrub did not fit our sampling criteria we chose the next closest shrub until we found an appropriate shrub.

We used canopy-intercept to determine aerial cover of *E*. *elymoides* and *P*. *spicata* along four transects that radiated from each shrub base. At each shrub (780 across all sites), we sampled transects in each cardinal direction. On each transect the “canopy” zone extended from shrub base to canopy dripline. When this distance was zero (in the case of asymmetric shrubs), no canopy sampling occurred. A shrub interspace was the region between two large shrubs of any species and in our study could be occupied by herbaceous vegetation and/or very small shrubs. The “interspace” zone extended from the canopy dripline to either a) the mid-point between canopy dripline and the nearest neighboring shrub canopy or b) 200 cm if the nearest neighboring shrub was more than 300 cm away. In cases of overlapping canopies only canopy measurements were taken for that transect. Due to the patchy growth form of *P*. *secunda* at our study sites, its aerial cover was visually estimated to the nearest 1% in 20 cm x 20 cm quadrats placed in the canopy (midpoint of canopy zone) and interspace (at both midpoint and end of the interspace) microsites (S1 Fig).

We also assessed *E*. *elymoides* and *P*. *spicata* densities within a 40 cm wide belt transect along each of the four transects which extended from shrub base to nearest shrub canopy or 200 cm (see above). A third microsite (edge), extended from 10 cm inside to 10 cm outside the canopy dripline (canopy and interspace zones were adjusted accordingly). When no canopy existed (e.g., an asymmetrical shrub), the interspace on that transect extended from shrub base to 200 cm or the midpoint to the nearest neighboring shrub canopy (whichever came first).

For each *E*. *elymoides* and *P*. *spicata* plant recorded in our density counts, we also measured its basal width and height. Basal width provides a measure of plant size that is not affected by current year grazing whereas height reflects both plant size and effects of current year grazing and precipitation. For each plant we also recorded evidence of whether it had been grazed (yes/no). We also recorded flowering (yes/no) as an assay of potential for reproduction. For this measure, we randomly selected *E*. *elymoides* and *P*. *spicata* plants at 10 sites between May 11 and July 1, 2014 (283 canopy / 249 interspace plants for *E*. *elymoides*, and 79 canopy / 98 interspace for *P*. *spicata*) (S1 Table). Due to difficulty in distinguishing *P*. *secunda* individuals, we did not record densities, sizes, height and grazing for *P*. *secunda*.

We characterized general vegetation conditions at each of the 32 field sites along the three 50-m focal shrub selection line transects described above. Following the methods of Herrick et al. [53], we assessed *Bromus tectorum*, perennial grass, annual forb and perennial forb cover, as well as bare ground with line-point intercept; basal and canopy gap width of perennial vegetation; and densities of cattle dung, perennial grasses, and *A*. *tridentata* shrubs in 2m wide belt transects.

### Data Analysis

We used sites as replicates in our analyses (n = 29 for *P*. *secunda*; n = 27 for *E*. *elymoides*; n = 10 for *P*. *spicata* cover; n = 14 for *P*. *spicata* density), pooling shrub-level means. Sample sizes differed among species and response variables because cover and density were too low at some sites to be included in our analyses. We used separate (i.e., one analysis per species per response variable) mixed-effects ANCOVA models with interaction terms to model the responses of perennial grasses to canopy vs. interspace microsites over a continuous rainfall gradient. Our response variables for nine main mixed-effects ANCOVA model analyses were site levels means for cover of *P*. *secunda*, *E*. *elymoides*, and *P*. *spicata*; density of *E*. *elymoides* and *P*. *spicata*; height of *E*. *elymoides* and *P*. *spicata*; and basal width of *E*. *elymoides* and *P*. *spicata*. The categorical predictor variable for all analyses was shrub microsite (canopy or interspace), and the continuous covariate was site-level PRISM rainfall estimate. Our model included a fixed microsite*rainfall interaction term, and the random effects were site and site*rainfall. The analysis for *E*. *elymoides* and *P*. *spicata* density also included “edge”, a third level of shrub microsite. Since preliminary analyses confirmed there was no year effect on percent cover and density of perennial grasses, we combined field data from 2012, 2013, and 2014. All analyses were conducted with SAS 9.3 [54] using the PROC GLIMMIX procedure with the Kenward-Roger method for calculating degrees of freedom.

We used t-tests to make pairwise comparisons and test if regression slope coefficients of the rainfall*microsite interaction term differed among sagebrush microsites (canopy, edge and interspace). We also used t-tests to compare canopy and interspace values for perennial grass cover and density values; when canopy > interspace, we inferred facilitation, and when canopy < interspace we inferred competition. We centered rainfall values around a mean of 0 to avoid obtaining negative extrapolative cover values for lower rainfall. To achieve residual homogeneity of variance and assumptions of normality we log-transformed *E*. *elymoides* cover data and square-root transformed *E*. *elymoides* density data. *Pseudoroegneria spicata* cover and density data were analyzed with beta and lognormal distributions, respectively.

We used a mixed logistic regression model to determine if the proportion of grazed plants differed among canopy, edge, and interspace sagebrush microsites for *P*. *spicata* and *E*. *elymoides*. For each species our response variable was the number of grasses grazed divided by the total number of grasses in each microsite (canopy, edge, or interspace). The model was run using PROC GLIMMIX [54], and pairwise comparisons were obtained using the LSMEANS statement within the main analysis for each grass species. We used a logistic regression model to determine if the proportion of flowering *E*. *elymoides* and *P*. *spicata* grasses differed among canopy, edge, and interspace microsites. The two separate analyses were conducted using PROC LOGISTIC; we applied the Firth adjustment for small sample sizes [55]. For each species the response variable was the number of grasses flowering divided by the total number of grasses in each microsite (canopy, edge, or interspace). For all four analyses, counts were pooled across sampling sites, which were the replicating factors and random variables in the model (n = 26 and n = 13 for *E*. *elymoides* and *P*. *spicata*, respectively).

We examined variables associated with general site conditions to explore broadscale patterns among rainfall and other site-level covariates. We examined bi-variate (Pearson) correlations between biologically meaningful covariates and cover of focal species in canopy and interspace microsites using PROC CORR [54]. We did this as an exploratory analysis to determine if rainfall was correlated with the site covariates we measured (S2 File). We did not perform additional model selection procedures due to the limited number of sites (i.e., replicates) in the study.

### Ethics Statement

Our non-destructive sampling did not require special permissions, and our study did not involve any endangered species.

## Results

### Cover

**Fig 3:**
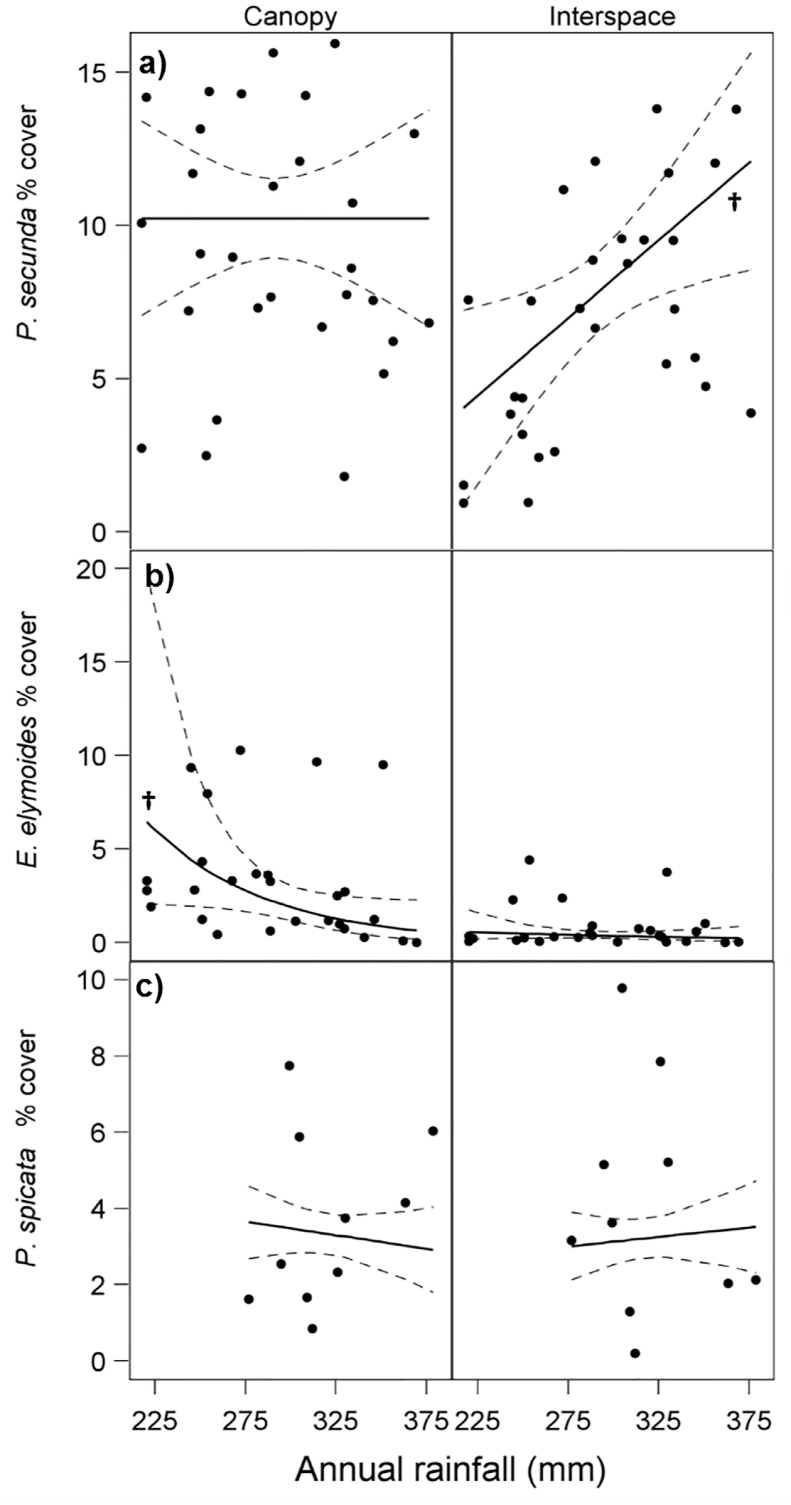
Mean percent cover and predicted regression lines (with 95% confidence bands) for a) *P*. *secunda* (n = 29 sites), b) *E*. *elymoides* (n = 27 sites) and c) *P*. *spicata* (n = 10 sites). Regression lines with slope significantly different from zero are denoted with (†). Sites are distributed across a rainfall gradient, and at each site cover was assessed for two microsites (shrub canopy and interspace). *E*. *elymoides* and *P*. *spicata* data are back-transformed.

We found a significant effect of a microsite*rainfall interaction on mean *P*. *secunda* aerial cover (Table 1; Fig 3a; S1 File), suggestive of a facilitative effect of *A*. *tridentata* canopies on *P*. *secunda* at low to moderate rainfall levels. In shrub interspaces, mean percent cover of *P*. *secunda* increased significantly from 4% at the lowest to 13% at the highest annual rainfall (*t*_*27*_ = 2.7, p = 0.01; Fig 3a). However, in sagebrush canopy microsites, there was no evidence to conclude that percent cover of *P*. *secunda* changed over increasing rainfall values (t_27_ = 0.0, p = 1.0; Fig 3a). Percent cover of *P*. *secunda* in shrub canopies was significantly greater than that of interspaces at low (25^th^ percentile, 249mm) to medium (50^th^ percentile, 278 mm) rainfall levels (t_24.4_ = 5.1, p < 0.0001, and t_24.4_ = 3.8, p = 0.0008, respectively). Differences were no longer significant at the 75^th^ percentile of annual rainfall (310 mm) (t_24.41_ = 0.51, p = 0.61).

**Table 1:** Type 3 fixed effects estimates for *P*. *secunda*, *E*. *elymoides*, and *P*. *spicata* analyses. F statistics (F), degrees of freedom (df), and p values (p) are given for each of the 3 main fixed effects: rainfall, sagebrush microsite (canopy, edge, interspace), and microsite*rainfall interaction.

			rainfall	microsite	microsite * rainfall
**Cover (%)**	*P*. *secunda* (n = 29)	F	2.08	13.34	12.78
		df	1,15.8	1,24.41	1,24.1
		p	0.17	0.001	0.002
	*E*. *elymoides* (n = 27)	F	2.38	63.07	3.98
		df	1,17.91	1,25.52	1,25.52
		p	0.14	<0.0001	0.06
	*P*. *spicata* (n = 14)	F	0.19	0.02	1.77
		df	1,7.19	1,7.22	1,7.37
		p	0.68	0.88	0.22
**Density (#/m** ^**2**^ **)**	*E*. *elymoides* (n = 27)	F	3.15	29.64	4.1
		df	1,11.86	2,38.26	2,38.26
		p	0.1	<0.0001	0.02
	*P*. *spicata* (n = 13)	F	4.58	0.07	0.34
		df	1,12	2,24	2,24
		p	0.05	0.93	0.72
**Height (cm)**	*E*. *elymoides* (n = 26)	F	0.11	40.64	0.07
		df	1,24	2,47	2,47
		p	0.75	<0.0001	0.93
	*P*. *spicata* (n = 13)	F	0.84	1.61	1.18
		df	1,11	2,18	2,18
		p	0.23	<0.0001	0.38
**Basal width (cm)**	*E*. *elymoides* (n = 26)	F	43.13	7.31	1.5
		df	1,24	2,47	2,47
		p	<0.0001	0.002	0.23
	*P*. *spicata* (n = 13)	F	2.86	7.3	2.14
		df	1,11	2,18	2,18
		p	0.12	0.005	0.15

There was a strong effect of shrub microsite on *E*. *elymoides* cover which was significantly greater in canopy versus interspace microsites over all rainfall levels (Table 1; S1 File). However, the effect appeared to be stronger at lower annual rainfall as evidenced by a marginally significant (p = 0.06) effect of the interaction between shrub microsite and rainfall on mean *E*. *elymoides* cover (Table 1; Fig 3b). Cover of *E*. *elymoides* decreased significantly in shrub canopies from about 5% at low rainfall levels to less than 1% at the highest rainfall levels (t_23.46_ = -2.13, p = 0.04). *Elymus elymoides* cover did not change significantly over rainfall values in interspace microsites (t_23.46_ = -0.68, p = 0.45). In contrast to *P*. *secunda* and *E*. *elymoides*, there was no significant effect of either microsite or rainfall on *P*. *spicata* cover (Table 1; Fig 3c; S1 File).

### Density

**Fig 4:**
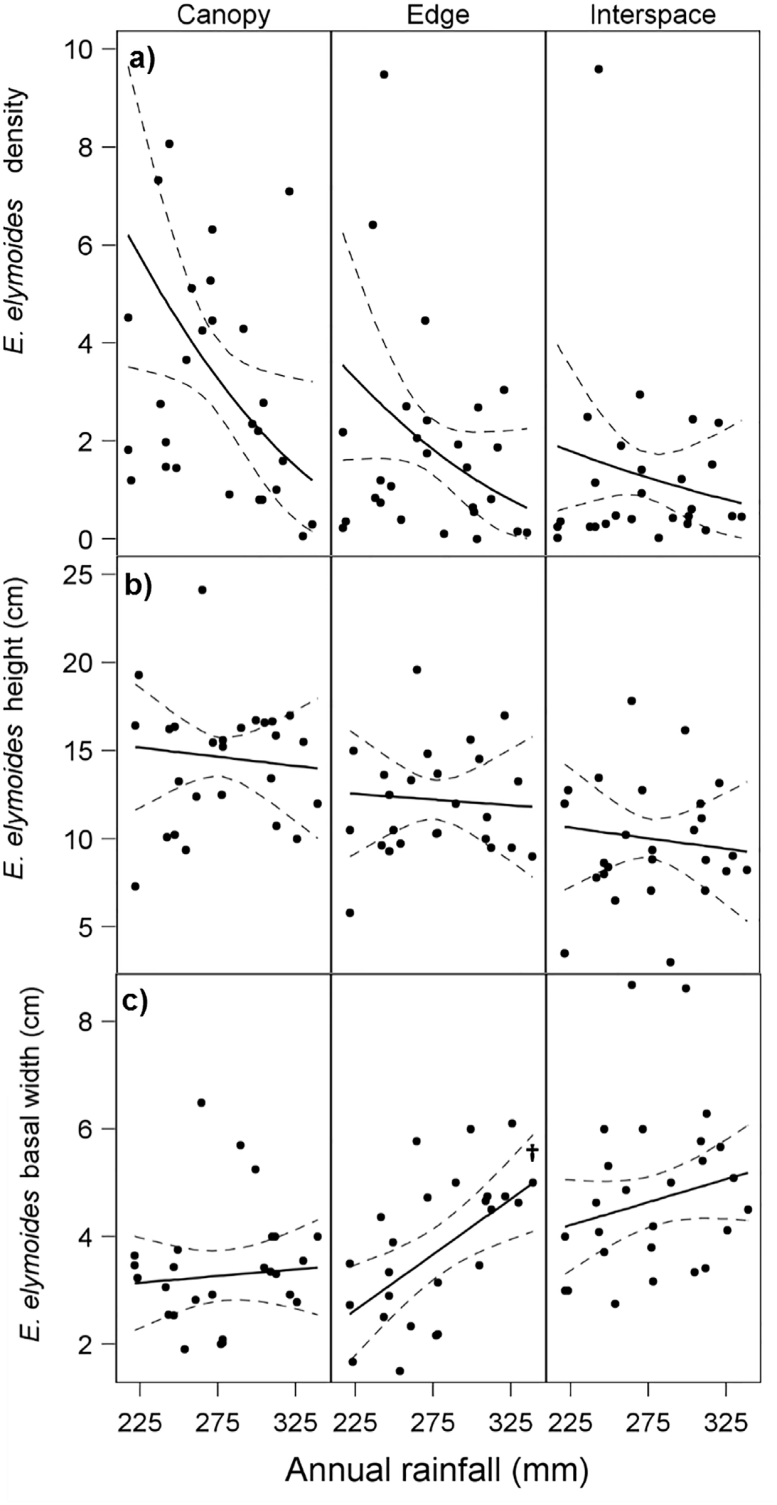
Site means and predicted regression lines (with 95% confidence bands) for *E*. *elymoides* for a) density (number of plants / m^2^), b) height (cm), and c) basal width (cm) across rainfall and shrub microsites. Regression lines with slope significantly different from zero are denoted with (†). Sites are distributed across a rainfall gradient, and at each site cover was assessed for three microsites (shrub canopy, edge, and interspace).

**Fig 5:**
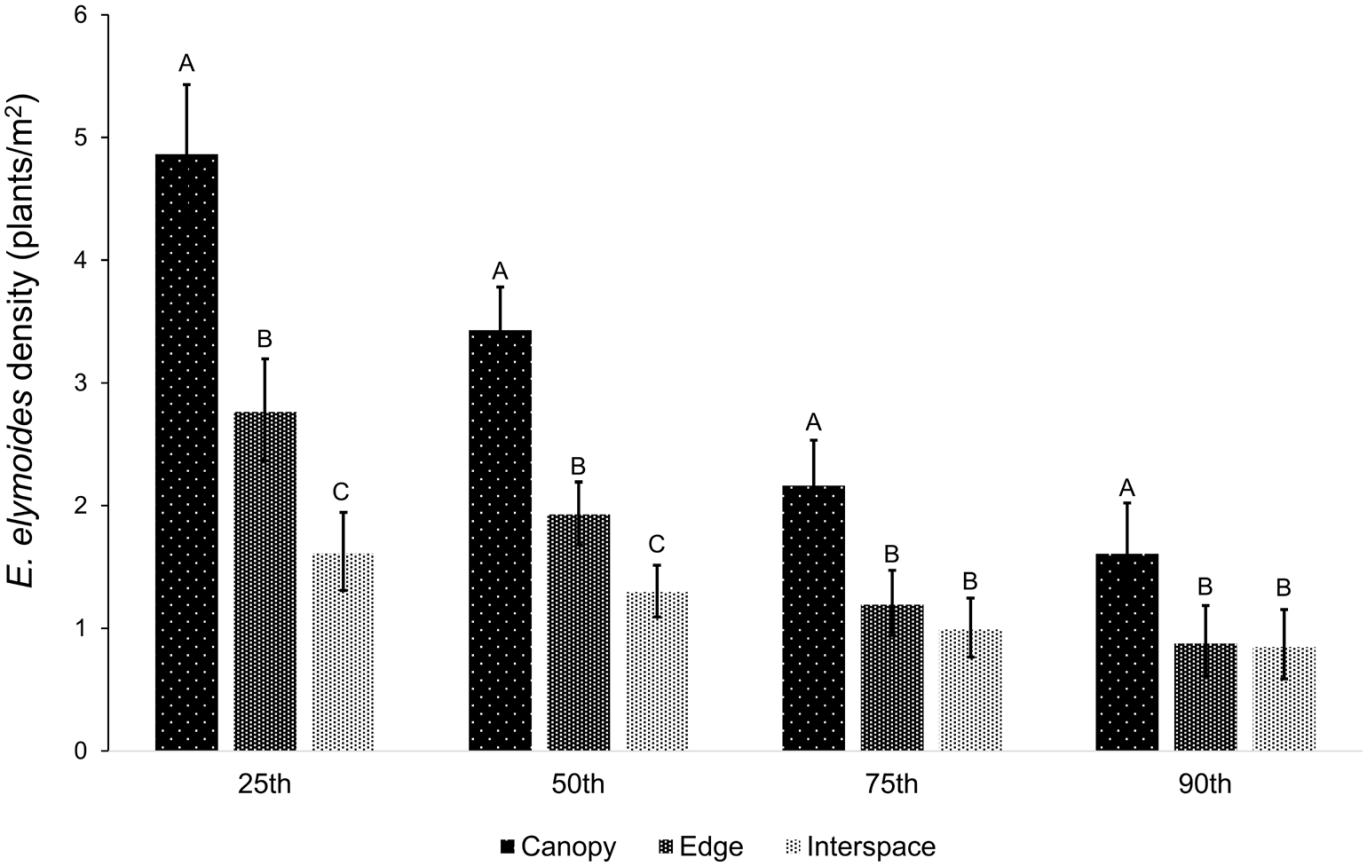
Least square means for *E*. *elymoides* density (plants/m^2^) at 25^th^, 50^th^, 75^th^, and 90^th^ percentile values of rainfall. Error bars represent standard error of the mean; different letters denote means that differ significantly from one another within each percentile of rainfall.

**Fig 6:**
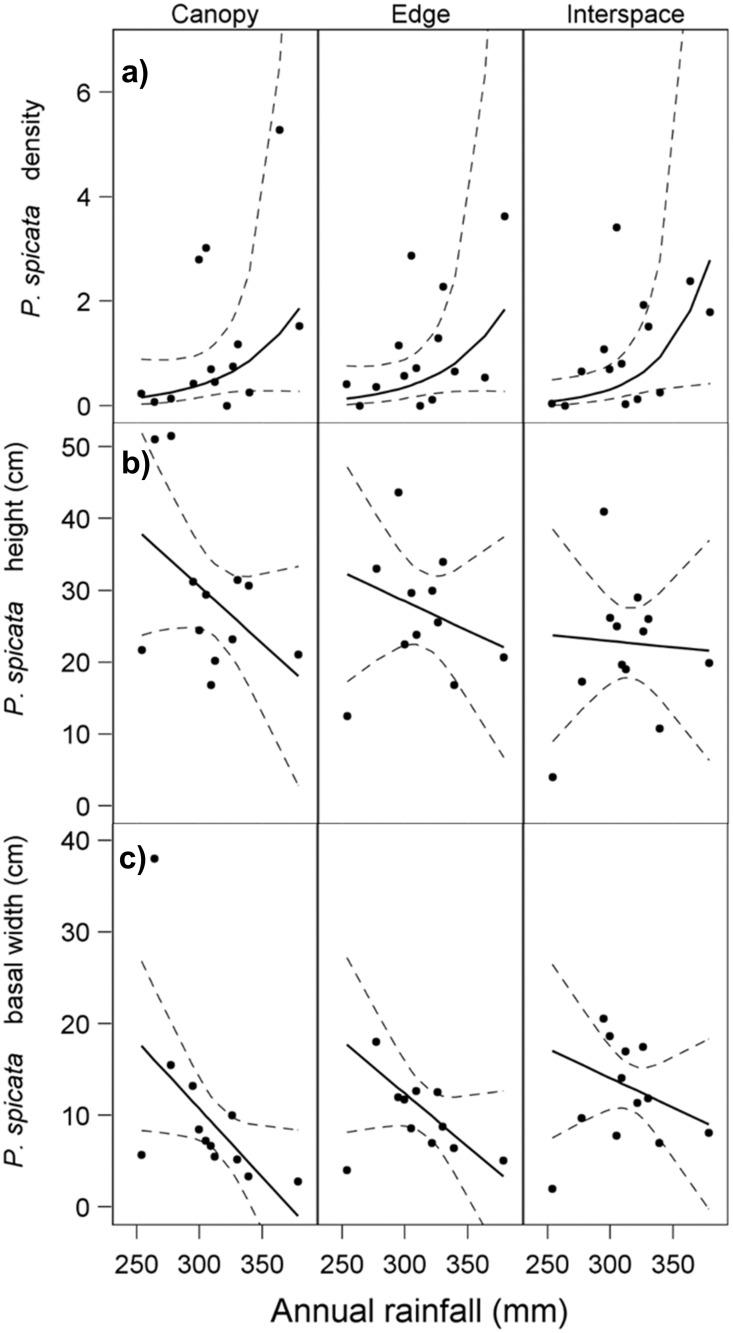
Site means and predicted regression lines (with 95% confidence bands) for *P*. *spicata* for a) density (number of plants / m^2^), b) height (cm), and c) basal width (cm) across rainfall and shrub microsites. All regression lines were not significantly different from 0. Sites are distributed across a rainfall gradient, and at each site cover was assessed for three microsites (canopy, edge, and interspace).

*Elymus elymoides* density results were consistent with a pattern of a stronger *A*. *tridentata* facilitative effect at lower rainfall levels. We found a significant interaction between shrub microsite and rainfall for *E*. *elymoides* mean densities (Table 1; Fig 4a; S1 File). The slopes in both canopy and edge microsites were significantly more negative (i.e., had higher densities at lower rainfall) than in interspace microsite (canopy vs. interspace: t_49.5_ = -3.14, p = 0.003; edge vs. interspace: t_49.5_ = 2.02, p = 0.05; Fig 4a). Differences in *E*. *elymoides* densities were significant for all pairwise microsite comparisons up to the 90^th^ percentile of the rainfall gradient (Fig 5). *Pseudoroegneria spicata* density responded significantly only to rainfall, increasing with increasing rainfall (Table 1; Fig 6; S1 File).

### Plant size

**Fig 7:**
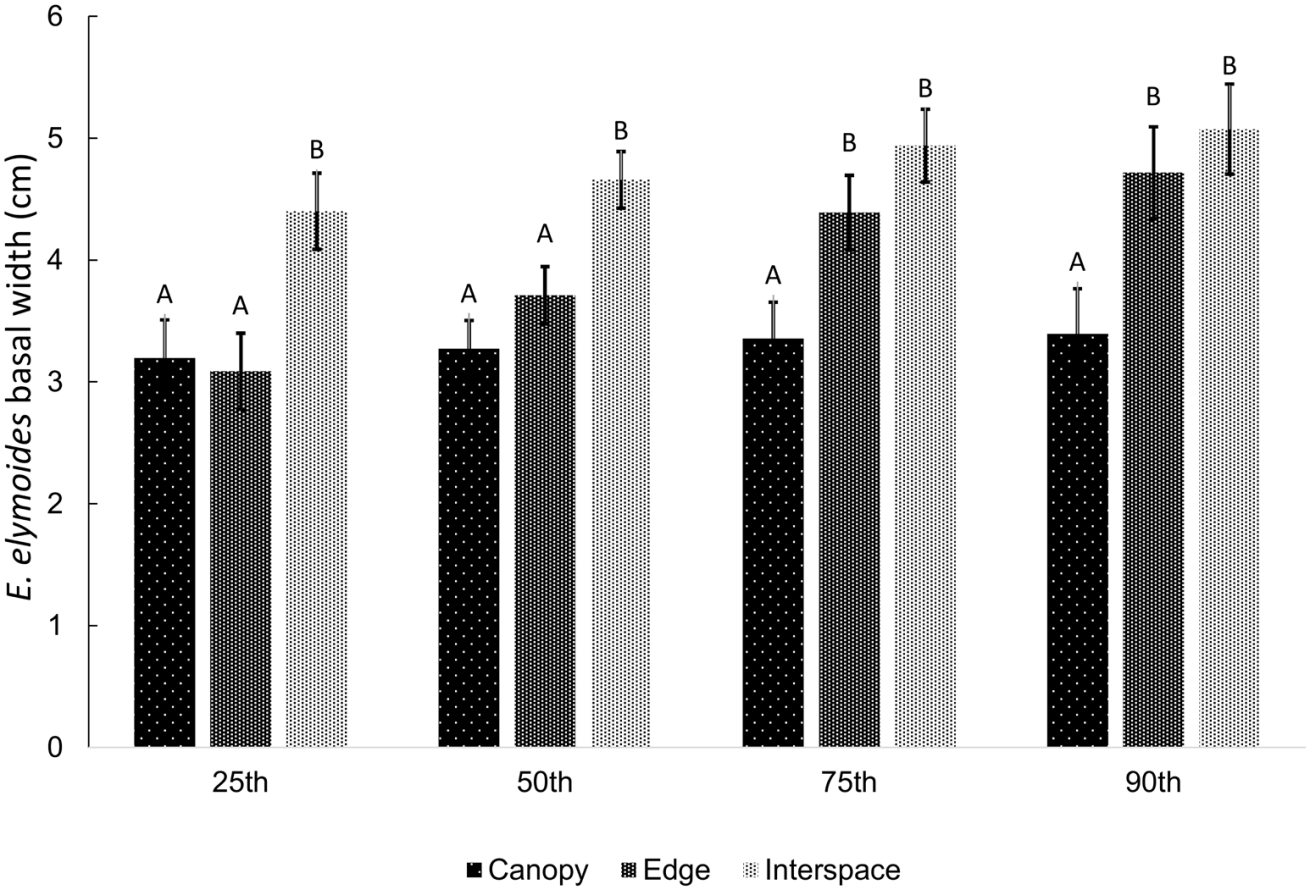
Least square means for *E*. *elymoides* basal width (cm) at 25^th^, 50^th^, 75^th^, and 90^th^ percentile of rainfall. Error bars represent standard error of the mean; different letters denote means that differ significantly from one another within each percentile of rainfall.

Height of *E*. *elymoides* (n = 26 sites) was greater in canopy than interspace microsites across all rainfall levels (Table 1, Fig 4b; S1 File); mean heights were as follows: canopy $\bar{x}=\;14.6\text{cm }(\sigma_{\bar{x}}=\;0.55)$, edge $\bar{x}=\;12.2\text{cm }(\sigma_{\bar{x}}=\;0.55)$, interspace $\bar{x}=\;10\text{cm }(\sigma_{\bar{x}}=\;0.93)$. Mean heights differed significantly for all pairwise microsite comparisons (canopy vs. interspace: t_47_ = 9.01, p < 0.0001; canopy vs. edge: t_47_ = 4.64, p < 0.0001; edge vs. interspace: t_47_ = 4.24, p = 0.0001; Fig 4b). Mean *E*. *elymoides* basal width (n = 26 sites) followed the opposite pattern; basal width was greatest in interspace microsites, followed by edge and canopy (canopy $\bar{x}=\;3.27\text{cm }[\sigma_{\bar{x}}=\;0.23]$, edge $\bar{x}=\;3.72\text{cm }[\sigma_{\bar{x}}=\;0.23]$, interspace $\bar{x}=\;4.66\text{cm }[\sigma_{\bar{x}}=\;0.23]$; Table 1; Fig 4c, Fig 7; S1 File). In addition, rainfall had an increasing effect on mean basal width for all microsites (Table 1), although the increase in basal width was significant only for edge microsites (t_41.97_ = 3.13, p = 0.0032; Fig 4c) and was steeper than canopy slopes (t_47.21_ = 2.72, p = 0.01). Mean basal widths generally differed significantly for canopy-interspace and edge-interspace microsite comparisons up to the 90^th^ percentile of rainfall (Fig 7).

Neither sagebrush microsite nor rainfall significantly affected *P*. *spicata* height (Table 1; Fig 6b; S1 File), although average height showed a trend of being highest in canopy microsites, followed by edge and interspace microsites: canopy $\bar{x}=\;28.8\text{cm }(\sigma_{\bar{x}}=\;2.4)$, edge $\bar{x}=\;27.6\text{cm }(\sigma_{\bar{x}}=\;2.5)$ interspace $\bar{x}=\;22.8{\text{cm (σ}}_{\bar{x}}\text{= 2.3)}$. *Pseudoroegneria spicata* basal width (n = 13 sites) was significantly affected only by microsite (Table 1) and was greatest in interspace microsites, followed by edge and canopy (canopy $\bar{x}=\text{ 9.13cm, }{\text{σ}}_{\bar{x}}\text{= 1.28}$; edge $\bar{x}=\text{ 11.2cm, }{\text{σ}}_{\bar{x}}\text{= 1.3}$; interspace $\bar{x}=\text{ 13.4cm, }{\text{σ}}_{\bar{x}}\text{= 1.24}$; Fig 6c; S1 File).

### Grazing effects and reproduction

The proportion of grazed grasses differed significantly among microsites for both *E*. *elymoides* and *P*. *spicata* (F _2,49_ = 75.9, p <0.0001; F _2,14_ = 75.9, p < 0.0001, respectively; S1 File). The proportion of grazed *E*. *elymoides* and *P*. *spicata* grasses was significantly greater in interspace vs. canopy microsites (t_49_ = -11.98, p < 0.0001; t_14_ = -3.09, p = 0.02, respectively), as well as interspace vs. edge microsites (t_49_ = -6.27, p < 0.0001; t_14_ = -2.68, p = 0.04, respectively).

The proportion of reproducing grasses did not differ significantly among microsites for either *E*. *elymoides* or *P*. *spicata* (Wald Chi-sq_2_ = 0.38, p = 0.84 and Wald Chi-sq_2_ = 0.79, p = 0.67; S1 File).

### Site characteristics and correlations

*Bromus tectorum* cover was negatively correlated with sagebrush density and positively correlated with perennial basal gap size (S1 File; S2 File). However, *Bromus tectorum* cover was not significantly correlated with cover of *E*. *elymoides*, *P*. *spicata*, or *P*. *secunda* in either sagebrush canopy or interspace microsites (S2 File). Dung density (our proxy of recent grazing intensity) was not significantly correlated with *E*. *elymoides*, *P*. *spicata*, or *P*. *secunda* in either sagebrush canopy or interspace microsites (S2 File). Several site variables (canopy gap size, basal gap size, bare ground cover, *Bromus tectorum* cover, annual forb cover, perennial forb cover and overall perennial grass cover) were correlated with rainfall (S2 File).

## Discussion

We found that cover of two native perennial grasses, *P*. *secunda* and *E*. *elymoides*, was strongly and positively associated with *A*. *tridentata* sub-canopy microsites at the lowest annual rainfall levels. These patterns are suggestive of net facilitative plant relationships in more stressful conditions (low annual rainfall) shifting to more neutral or competitive relationships in mesic conditions (high annual rainfall). These results, from across a regional rainfall gradient, provide support for the generality of the stress gradient hypothesis [39] and the prediction that stronger facilitation occurs in more stressful environmental conditions [11, 39, 56]. We suggest that sub-canopy microsites could be advantageous microsites for restoration plantings of *P*. *secunda* and *E*. *elymoides* in moisture-limited areas of the Great Basin. Lack of evidence for positive spatial associations between *A*. *tridentata* and the third grass species, *P*. *spicata*, however, also indicates the importance of considering species-specific relationships [57]. Our results are generally consistent with, but much broader in geographical scope, than previous work in northwestern portions of the Great Basin that has shown positive relationships between *A*. *tridentata* and native perennial grasses under stressful, moisture-limited conditions [7, 37].

### Shrub-grass patterns

We identified distinctly different facilitative patterns for the different grass species. At high rainfall, the net positive effect of *A*. *tridentata* on cover of the shallow-rooted grass, *P*. *secunda*, shifted to neutral, likely due in part to increased competition with *A*. *tridentata* and other herbaceous vegetation for soil moisture. Although sub-canopy cover of the medium-rooted grass, *E*. *elymoides*, decreased with increasing rainfall, *A*. *tridentata* still had a net facilitative effect on *E*. *elymoides* cover over almost the entire range of annual rainfall. Cover of the deep-rooted grass, *P*. *spicata*, was unaffected by proximity to *A*. *tridentata* nurse shrubs due to either a lack of nurse plant effect or low sample size. Our results are consistent with Davies et al. [7], who found increased *E*. *elymoides* cover under *A*. *tridentata* canopies at a drier site but no differences in sub-canopy *P*. *spicata* cover between dry and mesic sites. Our results for all three grass species contrast with those reported by Reisner et al. [33] who found evidence for net competitive shrub-grass relationships for *P*. *secunda* and *E*. *elymoides* in wetter conditions and positive relationships at drier sites for *P*. *spicata*. Our study encompassed a greater geographical extent and more site variability than Reisner et al. [33], which may explain our contrasting results.

Higher *Elymus elymoides* cover in sub-canopy microsites was driven by higher plant density in those microsites, rather than bigger plant sizes. Both *E*. *elymoides* and *P*. *spicata* sub-canopy microsite plants were taller and had smaller basal widths than those growing in edge or interspace microsites. The narrow, tall stature of sub-canopy plants may be a growth response to the increased ratio of far to near-red light found in sub-canopy conditions [58], or it could be due to increased inter- or intra-specific competition among adult plants for other resources [59, 60]. Alternatively, interspace plants may have been shorter due to grazing (see *Mechanisms* section below). Although reduced plant size could reduce fitness, we found that flowering was not reduced in sub-canopy microsites. Together our size and reproduction results suggest that targeting restoration plantings in sub-canopy microsites might yield smaller plants but not at the expense of establishing a self-sustaining (i.e., reproductive) population.

### Mechanisms

*Poa secunda* appears to benefit from growing in *A*. *tridentata* canopies at lower rainfall levels, perhaps because it is short-rooted and benefits from hydraulic lift from *A*. *tridentata* [61]. However, its shallower root system may be sufficient when site moisture is higher, thereby explaining its neutral association with *A*. *tridentata* at higher rainfall. Additionally, the benefits of sub-canopy microsites might be limited except under the driest conditions because *P*. *secunda* enters dormancy sooner than most Great Basin perennial grasses, [62] enabling it to largely avoid drought during the summer months [46, 62]. In contrast, *E*. *elymoide*s generally has a deeper root system than *P*. *secunda* but continues to grow later into the summer months. As moisture stress increases over the summer, *E*. *elymoides* may increasingly benefit from the improved microsite conditions (e.g., lower temperatures, reduced solar radiation, or increased organic matter) in shrub sub-canopies, even at higher rainfall levels. The deep-rooted *P*. *spicata*, on the other hand, can survive under a very wide range of moisture conditions [63], which may allow it to persist in both sub-canopy and interspace microsites. Understanding these types of species- and growth form-specific patterns is key toward reconciling opposing views on the importance and nature of the stress-gradient hypothesis [57]. To clarify the importance of rooting depth and species biology in shrub-grass relationships, we suggest that future studies include multiple species of each growth form.

Reproductive strategies and seed dispersal patterns also may influence plant spatial patterns. For example, *Elymus elymoides*, which only reproduces via seed, may be more likely to establish near or under *A*. *tridentata* canopies because shrubs can act as seed traps, simultaneously limiting the dispersal of seeds dropped within shrub canopies and trapping windblown seeds at their periphery [64]. The large seedheads of *E*. *elymoides* were observed in great numbers under sagebrush canopies (MFH *pers*. *obs*). In contrast, because *P*. *spicata* produces less seed and propagates mainly by tillering [48], distribution of this species may depend more on location of parent plants than seed dispersal (and trapping by *A*. *tridentata*). Small *P*. *secunda* seeds may be trapped by microtopography of the soil surface [65] of either shrub canopies or interspaces.

Herbivory also can mediate plant facilitation and even intensify abiotically-driven facilitation [9, 21]. *Artemisia tridentata* sub-canopy microsites may provide physical protection against large herbivore grazing [9], particularly for palatable grasses [66]. In our study all sites had been spring grazed at least once in the past five years, and *E*. *elymoides* and *P*. *spicata* plants, both of which are palatable to livestock [47, 50], were grazed more in interspace than sub-canopy microsites. However, we did not find our livestock grazing intensity covariate (dung density) to be significantly correlated with *E*. *elymoides* or *P*. *spicata* cover or rainfall. But, because perennial grass densities likely decrease with decreasing rainfall (as suggested by a significant negative correlation between rainfall and inter-plant gap size), we cannot rule out the possibility that rainfall and relative grazing stress covary. That is, if livestock activity (as measured by dung density) was indeed not lower at lower rainfall sites, there may be greater relative grazing pressure on fewer plants at those drier sites. Therefore, the shrub-grass associations we observed may have been driven in part by associational defense offered to grasses by growing in shrub canopies, away from grazers. We are conducting further work to explore whether canopy-interspace patterns for these two grasses differ between low and high grazing intensity sites.

### Applications to ecological restoration and future research needs

We found that naturally occurring *P*. *secunda* and *E*. *elymoides* plants were associated with *A*. *tridentata* canopy microsites, particularly in drier areas. These patterns suggest that exploiting sub-canopy microsites for restoration seedings or plantings in Great Basin sagebrush communities could improve plant establishment, growth, or survival (or some combination thereof) sensu [19, 67]. Although *E*. *elymoides* distributions may be driven at least partially by trapping of seeds and increased propagule pressure in shrub understories, our results clearly show that plants persist to maturity in understory microsites. Future studies examining responses of plants of different growth forms and at multiple life stages would help clarify the mechanisms behind the patterns we observed.

Our results are most applicable to areas of the Great Basin that contain shrub overstories but lack a robust perennial understory and are at risk of invasion by undesirable annuals. Typically, however, restoration efforts in the Great Basin target areas where sagebrush canopies are no longer intact such as burned areas. Our results highlight the potential utility of shrubs to serve as nurse plants in restoration settings and provide important ecosystem functions.

Based on the results of our study we suggest that land managers in the Great Basin should consider the utility of a nurse plant approach as way to improve the resilience of sagebrush plant communities to disturbance, as well as resistance to invasive species (sensu [32]). To translate our results into practice will require controlled experimentation that explicitly investigates, across species and rainfall levels, canopy microsite effects on multiple plant life stages, from germination to establishment, survival, and long-term population viability (e.g., reproductive potential and dispersal/establishment beyond sub-canopy microsites). Nonetheless, our results highlight that, when planning and prioritizing restoration activities across the Great Basin, average annual rainfall level may indicate where positive plant associations can be expected to occur.

## Supporting Information

S1 Table Site characteristics for sites sampled during 2012, 2013, and 2014 field seasons. PRISM rainfall refers to annual rainfall values predicted by PRISM data [36]. ESD rainfall refers to annual rainfall ranges based upon ecological site descriptions (Natural Resources Conservation Service 2006). MLRA refers to Major Land Resources Areas. ARTR refers to the focal shrub species *Artemisia tridentata* ssp. *wyomingensis*.
(DOCX)

S1 Fig Sampling scheme for *Poa secunda* as seen from above, depicting a sagebrush canopy and transect extending from the base. Numbers correspond to placement of 20 cm x 20 cm quadrats for estimating percent cover of *P*. *secunda*. 1) Canopy: quadrat placed at approximate midpoint of canopy region; 2) Interspace: quadrat placed at approximate midpoint of transect; 3) Interspace: quadrat placed at end of transect.
(DOCX)

S1 File Site level data for environmental covariates and focal species cover, density, basal width, and height means. Means were been averaged over shrub-level (subsample) data values.
(DOCX)

S2 File Pairwise correlations for site-level variables and focal grass species cover for canopy and interspace microsites. P values test the null hypotheses that Pearson’s pairwise correlations between 2 variables = 0 versus the alternative hypothesis that Pearson’s pairwise correlations between 2 variables ≠ 0.
(DOCX)
