# Supplementary material for: Grass-Shrub Associations over a Precipitation Gradient and Their Implications for Restoration in the Great Basin, USA
Source: PLoS One. 2015 Dec 1;10(12):e0143170. doi: 10.1371/journal.pone.0143170 (PMC4666403; doi:10.1371/journal.pone.0143170)
Supplement: S1 Table — PRISM rainfall refers to annual rainfall values predicted by PRISM data [36]. ESD rainfall refers to annual rainfall ranges based upon ecological site descriptions (Natural Resources Conservation Service 2006). MLRA refers to Major Land Resources Areas. ARTR refers to the focal shrub species Artemisia tridentata ssp. wyomingensis. (DOCX) [file pone.0143170.s001.docx]

**S1 Table**. **Site characteristics for sites sampled during 2012, 2013, and 2014 field seasons**.

| Site | PRISM rainfall mm | ESD rainfall mm | MLRA | State | Year sampled | Latitude | Longitude | Mean ARTR height (cm) | Mean ARTR canopy width (cm) | Mean ARTR interspace length (cm) | Shrub density (per m^2^); no ARTR | ARTR density (per m^2^) |
| --- | --- | --- | --- | --- | --- | --- | --- | --- | --- | --- | --- | --- |
| J521DUX_02 | 221 | 203-254 | Humboldt Area | NV | 2012*● | 41.062982 | -118.45537 | 94.7 | 90.8 | 135.9 | 0.1 | 0.6 |
| Grasmere 5 | 241 | 178-254 | Owyhee High Plateau | ID | 2012,  2014*●○□ | 42.444377 | -115.84427 | 66.3 | 67.7 | 125.1 | 0.2 | 1.0 |
| X378AUX_01 | 243 | 203-254 | Humboldt Area | NV | 2012*● | 40.86218 | -118.10173 | 45.4 | 38.1 | 133.6 | 0.0 | 2.0 |
| Grasmere 3 | 249 | 178-254 | Owyhee High Plateau | ID | 2012,  2014*●○□ | 42.33215 | -115.84252 | 51.9 | 63 | 138.8 | 0.1 | 0.6 |
| z269aux01 | 250 | 203-305 | Snake River Plains | ID | 2012* | 42.93022 | -115.31045 | 60.2 | 61.1 | 132.9 | 0.0 | 0.9 |
| X022AUX_02 | 253 | 203-254 | Central Nevada Basin and Range | NV | 2012*●■ | 40.920339 | -114.53371 | 52.8 | 52.4 | 94.6 | 0.4 | 1.4 |
| Squaw | 254 | 254-330 | Owyhee High Plateau | ID | 2012*●■ | 43.430404 | -116.86181 | 44.9 | 43.6 | 106.6 | 0.0 | 1.4 |
| x428DUX_02 | 260 | 203-254 | Humboldt Area | NV | 2012*●■ | 40.485524 | -117.43795 | 53 | 54.5 | 111.2 | 0.6 | 1.1 |
| J489DUX_03 | 264 | 203-254 | Humboldt Area | NV | 2012*●■ | 41.26252 | -117.71296 | 55.6 | 44.5 | 105.5 | 0.2 | 1.1 |
| f555aux01 | 271 | 203-305 | Snake River Plains | ID | 2012*● | 42.366647 | -114.47114 | 61.1 | 62.6 | 124.8 | 0.0 | 0.0 |
| Dam | 277 | 203-305 | Owyhee High Plateau | ID | 2012*● | 42.206451 | -114.76649 | 57.9 | 69.8 | 111.9 | 0.0 | 0.7 |
| Palisade | 292 | 203-254 | Owyhee High Plateau | NV | 2012* | 40.53208 | -116.28477 | 56.1 | 59.2 | 137.5 | 0.0 | 1.0 |
| Bigfoot | 292 | 203-305 | Snake River Plains | ID | 2012,  2014*●○□ | 43.247636 | -116.25227 | 42.5 | 40.3 | 131.6 | 0.0 | 3.6 |
| Tuscarora | 305 | 254-305 | Owyhee High Plateau | NV | 2012, 2014*●○■□ | 41.59531 | -116.35237 | 95.8 | 92.3 | 140.5 | 0.1 | 0.4 |
| N245AUX_01 | 308 | 254-330 | Owyhee High Plateau | ID | 2012, 2014*●○■□ | 43.446 | -116.968 | 56.9 | 53.6 | 139.5 | 0.2 | 0.4 |
| X039DUX_03 | 312 | 203-254 | Owyhee High Plateau | NV | 2012*● | 41.1728 | -114.867 | 52.8 | 57.9 | 106.7 | 0.1 | 1.1 |
| Dugway east | 321 | 203-305 | Great Salt Lake | UT | 2012*● | 40.058312 | -112.70609 | 41.4 | 46.6 | 105.6 | 0.0 | 2.1 |
| F463aux_02 | 326 | 279-330 | Snake River Plains | ID | 2012, 2014*●○■□ | 43.279496 | -114.32595 | 49 | 56.4 | 127.8 | 0.1 | 0.3 |
| R157DUX_03 | 357 | 203-305 | Great Salt Lake | UT | 2012* | 40.2456 | -112.627 | 70.2 | 60.5 | 160.3 | 0.0 | 0.6 |
| WilsonHi | 310 | 254-305 | Owyhee High Plateau | NV | 2013*● | 41.662003 | -116.33469 | 61.4 | 61.1 | 134.4 | 0.6 | 0.8 |
| WilsonLow | 312 | 254-305 | Owyhee High Plateau | NV | 2013,  2014*●■○□ | 41.655902 | -116.31676 | 50.6 | 53.7 | 129.4 | 0.1 | 0.8 |
| AntelopeHi | 221 | 203-254 | Humboldt Area | NV | 2013*● | 41.30878 | -117.6917 | 84.7 | 91.9 | 124.5 | 0.1 | 0.5 |
| AntelopeLow | 223 | 203-254 | Humboldt Area | NV | 2013*● | 41.319994 | -117.68408 | 64.4 | 66.5 | 136.9 | 0.1 | 0.8 |
| DamHigh | 278 | 203-305 | Owyhee High Plateau | ID | 2013*● | 42.20115 | -114.76608 | 77.4 | 75.1 | 137.7 | 0.4 | 0.6 |
| DamLow | 278 | 203-305 | Owyhee High Plateau | ID | 2013*● | 42.196978 | -114.76697 | 31.9 | 39.8 | 113.7 | 0.7 | 1.9 |
| Elephant | 339 | 254-330 | Owyhee High Plateau | ID | 2013* | 43.373211 | -116.84716 | 65.6 | 70.2 | 146.2 | 0.8 | 0.2 |
| Buttelo | 330 | 254-330 | Owyhee High Plateau | ID | 2013*●■ | 43.37698 | -116.88564 | 69.5 | 63.9 | 142.6 | 0.1 | 0.0 |
| JackCrk | 246 | 254-330 | Owyhee High Plateau | ID | 2012*● | 42.463327 | -115.92876 | 62.6 | 54.3 | 147.5 | 0.0 | 1.1 |
| JackCrkh20 | 246 | 254-330 | Owyhee High Plateau | ID | 2013*● | 42.465466 | -115.91969 | 90 | 80.9 | 118.4 | 0.0 | 0.4 |
| F174DUX_01 | 277 | 254-330 | Owyhee High Plateau | ID | 2014○■□ | 42.102869 | -114.7864 | - | - | - | - | - |
| Q613_AUX02 | 378 | 330-457 | Great Salt Lake | UT | 2014○■□ | 39.667749 | -112.04157 | - | - | - | - | - |
| J527_AUX03 | 294 | 203-254 | Humboldt Area | NV | 2014○■□ | 41.436878 | -117.05473 | - | - | - | - | - |

**Symbol legend for measurements made:**

**P. secunda* cover

●*E. elymoides* density, cover, basal width, height, evidence of grazing

○*E. elymoides* flowering

■ *P. spicata* density, cover, basal width, height, evidence of grazing

□*P. spicata* flowering
