# Supplementary material for: Grass-Shrub Associations over a Precipitation Gradient and Their Implications for Restoration in the Great Basin, USA
Source: PLoS One. 2015 Dec 1;10(12):e0143170. doi: 10.1371/journal.pone.0143170 (PMC4666403; doi:10.1371/journal.pone.0143170)
Supplement: S1 Fig — Numbers correspond to placement of 20 cm x 20 cm quadrats for estimating percent cover of P. secunda. 1) Canopy: quadrat placed at approximate midpoint of canopy region; 2) Interspace: quadrat placed at approximate midpoint of transect; 3) Interspace: quadrat placed at end of transect. (DOCX) [file pone.0143170.s002.docx]

**S1 Figure.** Sampling scheme for *Poa secunda* as seen from above, depicting a sagebrush canopy and transect extending from the base.

Sagebrush canopy

3

2

1

Interspace
