# Supplementary material for: Grass-Shrub Associations over a Precipitation Gradient and Their Implications for Restoration in the Great Basin, USA
Source: PLoS One. 2015 Dec 1;10(12):e0143170. doi: 10.1371/journal.pone.0143170 (PMC4666403; doi:10.1371/journal.pone.0143170)
Supplement: S1 File — Means were been averaged over shrub-level (subsample) data values. (DOCX) [file pone.0143170.s003.docx]

Raw data used for analyses. The perennial grasses, *Elymus elymoides* (ELEL), *Poa secunda* (POSE), and *Pseudoroegneria spicata* (PSSP), are shown as mean percent cover in canopies (C) and interspaces (I) of *Artemisia tridentata* (ARTR). Annual rainfall is measured in inches/year. Density covariates are measured as # / m^2^. ARTR volume is measured in cm^3^. “Basal gap size” refers to average size (cm) of gaps between bases of perennial vegetation, while “perennial canopy gap size” refers to average size (cm) of gaps between canopies of perennial vegetation. All covariates with a “prp” suffix denote site-level proportions of ground cover and were obtained from line-point intercept sampling during site characterization. Blank cells indicate no data.

| Site | *ELEL* cover  (C) | *ELEL* cover  (I) | *POSE* cover (C) | *POSE* cover (I) | *PSSP*  Cover (C) | *PSSP*  Cover (I) | Annual rainfall | density cow dung pats | ARTR  density | ARTR volume | basal gap size | perennial canopy gap size | bare ground prp | annual forb prp | ARTR prp | perennial forb prp | perennial grass prp |
| --- | --- | --- | --- | --- | --- | --- | --- | --- | --- | --- | --- | --- | --- | --- | --- | --- | --- |
| J489 | 10.29 | 2.39 | 14.31 | 11.18 |  |  | 10.40 | 0.01 | 0.37 | 1.04 | 100.81 | 75.12 | 0.07 | 0.00 | 0.27 | 0.01 | 0.66 |
| X378 | 2.80 | 0.12 | 11.70 | 4.41 |  |  | 9.57 | 0.08 | 0.02 | 0.44 | 610.73 | 142.54 | 0.24 | 0.09 | 0.17 | 0.00 | 0.19 |
| antelopehi | 2.78 | 0.06 | 2.74 | 0.95 |  |  | 8.70 | 0.11 | 0.61 | 0.93 | 944.20 | 228.96 | 0.20 | 0.18 | 0.23 | 0.00 | 0.01 |
| antelopelo | 1.90 | 0.22 | 14.19 | 7.58 |  |  | 8.78 | 0.00 | 1.04 | 0.55 | 127.44 | 88.31 | 0.10 | 0.01 | 0.22 | 0.01 | 0.47 |
| bigfoot | 1.14 | 0.03 | 12.10 | 9.57 |  |  | 11.40 | 0.08 | 1.97 | 0.11 | 156.74 | 93.42 | 0.33 | 0.01 | 0.27 | 0.00 | 0.39 |
| buttelo | 9.50 | 1.02 | 6.21 | 12.04 | 3.75 | 5.22 | 12.99 | 0.15 | 0.64 | 0.34 | 71.30 | 53.19 | 0.08 | 0.00 | 0.16 | 0.05 | 0.63 |
| dam | 3.60 | 0.52 | 7.67 | 8.88 |  |  | 10.90 | 0.07 | 0.92 | 0.26 | 107.50 | 69.94 | 0.28 | 0.00 | 0.30 | 0.02 | 0.42 |
| damhi | 3.27 | 0.89 | 11.29 | 6.65 |  |  | 10.94 | 0.10 | 1.42 | 0.17 | 116.06 | 88.35 | 0.18 | 0.22 | 0.34 | 0.00 | 0.35 |
| damlo | 0.61 | 0.38 | 15.65 | 12.10 |  |  | 10.94 | 0.00 | 1.39 | 0.11 | 88.51 | 60.29 | 0.11 | 0.05 | 0.35 | 0.02 | 0.56 |
| dugway | 0.29 | 0.07 | 7.56 | 5.70 |  |  | 12.65 | 0.00 | 1.11 | 0.29 | 151.64 | 85.34 | 0.20 | 0.01 | 0.37 | 0.00 | 0.30 |
| elephanthi | 0.10 | 0.00 | 13.01 | 13.80 |  |  | 13.35 | 0.22 | 1.12 | 0.26 | 76.33 | 48.94 | 0.08 | 0.00 | 0.33 | 0.00 | 0.53 |
| f174 |  |  |  |  | 1.61 | 3.17 | 10.90 |  |  |  |  |  |  |  |  |  |  |
| f463 | 1.24 | 0.57 | 5.16 | 4.76 | 2.33 | 7.85 | 12.83 | 0.00 | 0.01 | 0.34 | 147.74 | 93.86 | 0.22 | 0.01 | 0.23 | 0.03 | 0.38 |
| f555 | 3.68 | 0.28 | 7.31 | 7.30 |  |  | 10.68 | 0.01 | 0.67 | 0.40 | 117.67 | 65.32 | 0.18 | 0.00 | 0.37 | 0.02 | 0.43 |
| gras3 | 7.97 | 4.41 | 2.49 | 0.96 |  |  | 9.80 | 0.09 | 1.02 | 0.29 | 399.33 | 501.55 | 0.46 | 0.06 | 0.21 | 0.00 | 0.20 |
| gras5 | 9.36 | 2.27 | 7.23 | 3.85 |  |  | 9.50 | 0.08 | 3.64 | 0.13 | 147.62 | 78.44 | 0.26 | 0.02 | 0.33 | 0.00 | 0.28 |
| j521 | 3.32 | 0.35 | 10.08 | 1.53 |  |  | 8.70 | 0.06 | 0.37 | 0.22 | 649.14 | 297.84 | 0.49 | 0.18 | 0.06 | 0.00 | 0.11 |
| j527 |  |  |  |  | 2.55 | 5.15 | 11.60 |  |  |  |  |  |  |  |  |  |  |
| jack | 1.25 | 0.27 | 13.15 | 4.38 |  |  | 9.70 | 0.00 | 1.06 | 0.21 | 189.55 | 113.43 | 0.37 | 0.01 | 0.36 | 0.00 | 0.24 |
| jackcrkh20 | 4.34 | 0.26 | 9.08 | 3.19 |  |  | 9.70 | 0.10 | 2.07 | 0.13 | 180.31 | 83.62 | 0.31 | 0.00 | 0.31 | 0.00 | 0.23 |
| n245 | 2.50 | 0.37 | 1.80 | 5.48 | 1.67 | 1.30 | 12.16 | 0.05 | 0.25 | 0.27 | 241.24 | 71.25 | 0.18 | 0.00 | 0.35 | 0.00 | 0.40 |
| palisade | 0.00 | 0.00 | 14.25 | 8.76 |  |  | 11.50 | 0.10 | 0.57 | 0.34 | 88.02 | 62.44 | 0.37 | 0.02 | 0.11 | 0.00 | 0.61 |
| q613 |  |  |  |  | 6.04 | 2.14 | 14.90 |  |  |  |  |  |  |  |  |  |  |
| r157 | 0.00 | 0.03 | 6.82 | 3.89 | 4.16 | 2.04 | 13.60 | 0.02 | 0.76 | 0.28 | 154.95 | 113.08 | 0.20 | 0.01 | 0.23 | 0.00 | 0.39 |
| squaw | 9.67 | 0.73 | 6.69 | 9.53 | 7.74 | 3.62 | 11.78 | 0.00 | 0.84 | 0.20 | 79.11 | 63.00 | 0.27 | 0.00 | 0.29 | 0.01 | 0.53 |
| tuscarora | 1.19 | 0.64 | 15.95 | 13.82 | 5.88 | 9.79 | 12.00 | 0.09 | 0.46 | 0.87 | 82.34 | 48.25 | 0.14 | 0.02 | 0.25 | 0.01 | 0.60 |
| wilsonhi | 1.00 | 0.31 | 7.74 | 11.72 |  |  | 12.20 | 0.19 | 0.78 | 0.60 | 58.80 | 46.66 | 0.05 | 0.04 | 0.15 | 0.19 | 0.47 |
| wilsonlo | 0.73 | 0.02 | 8.61 | 9.52 | 0.84 | 0.20 | 12.28 | 0.10 | 0.56 | 0.72 | 74.20 | 53.21 | 0.09 | 0.11 | 0.21 | 0.15 | 0.37 |
| x022 | 0.43 | 0.06 | 3.65 | 2.43 |  |  | 9.98 | 0.04 | 1.86 | 0.07 | 198.54 | 92.33 | 0.36 | 0.01 | 0.38 | 0.01 | 0.11 |
| x039 | 2.73 | 3.77 | 10.75 | 7.27 |  |  | 12.30 | 0.05 | 0.22 | 0.48 | 93.32 | 56.43 | 0.30 | 0.00 | 0.08 | 0.15 | 0.51 |
| x428 | 3.32 | 0.32 | 8.97 | 2.63 |  |  | 10.25 | 0.01 | 1.06 | 0.28 | 244.84 | 154.74 | 0.38 | 0.05 | 0.21 | 0.00 | 0.22 |
| z269 | 0.00 | 0.00 | 14.38 | 7.55 |  |  | 9.85 | 0.09 | 0.35 | 0.71 | 168.55 | 118.48 | 0.07 | 0.00 | 0.26 | 0.00 | 0.57 |
