# Supplementary material for: Grass-Shrub Associations over a Precipitation Gradient and Their Implications for Restoration in the Great Basin, USA
Source: PLoS One. 2015 Dec 1;10(12):e0143170. doi: 10.1371/journal.pone.0143170 (PMC4666403; doi:10.1371/journal.pone.0143170)
Supplement: S2 File — P values test the null hypotheses that Pearson’s pairwise correlations between 2 variables = 0 versus the alternative hypothesis that Pearson’s pairwise correlations between 2 variables ≠ 0. (DOCX) [file pone.0143170.s004.docx]

**Pairwise correlations, p values and sample sizes for site-level variables (first 10 columns) and focal grass species (last three columns) for a) canopy microsites and b) interspace microsites**

**a) Canopy microsites**

|  | **Perennial grass cover** | **ARTR density** | **rainfall** | **Bare ground cover** | **Cow pat density** | **Perennial basal gap size** | **Perennial canopy gap size** | **BRTE cover** | **Annual forb cover** | **Perennial forb cover** | **PSSP cover** | **POSE cover** | **ELEL cover** |
| --- | --- | --- | --- | --- | --- | --- | --- | --- | --- | --- | --- | --- | --- |
| **Perennial grass cover** | 1 | -0.24534 | 0.53948 | -0.59114 | 0.09454 | -0.73144 | -0.56569 | -0.03072 | -0.5135 | 0.22596 | 0.64134 | 0.51155 | 0.07317 |
| **p** |  | 0.1996 | 0.0025 | 0.0007 | 0.6257 | <.0001 | 0.0014 | 0.8743 | 0.0044 | 0.2385 | 0.1206 | 0.0046 | 0.706 |
| **n** | 29 | 29 | 29 | 29 | 29 | 29 | 29 | 29 | 29 | 29 | 7 | 29 | 29 |
| **ARTR density** | -0.24534 | 1 | -0.24538 | 0.19483 | 0.07388 | -0.18271 | -0.06664 | -0.37092 | -0.07 | -0.22806 | 0.57244 | -0.05043 | 0.19967 |
| **p** | 0.1996 |  | 0.1995 | 0.3112 | 0.7033 | 0.3428 | 0.7313 | 0.0476 | 0.7182 | 0.2341 | 0.1793 | 0.795 | 0.2991 |
| **n** | 29 | 29 | 29 | 29 | 29 | 29 | 29 | 29 | 29 | 29 | 7 | 29 | 29 |
| **rainfall** | 0.53948 | -0.24538 | 1 | -0.38556 | 0.19282 | -0.57647 | -0.47351 | -0.31555 | -0.36427 | 0.35837 | 0.31735 | -0.07657 | -0.19811 |
| **p** | 0.0025 | 0.1995 |  | 0.0389 | 0.3163 | 0.0011 | 0.0095 | 0.0785 | 0.0521 | 0.0563 | 0.3716 | 0.693 | 0.3029 |
| **n** | 29 | 29 | 32 | 29 | 29 | 29 | 29 | 32 | 29 | 29 | 10 | 29 | 29 |
| **Bare ground cover** | -0.59114 | 0.19483 | -0.38556 | 1 | -0.25645 | 0.36772 | 0.56662 | -0.34047 | 0.15225 | -0.32411 | 0.46552 | -0.24183 | 0.08483 |
| **p** | 0.0007 | 0.3112 | 0.0389 |  | 0.1793 | 0.0497 | 0.0014 | 0.0707 | 0.4304 | 0.0863 | 0.2925 | 0.2063 | 0.6617 |
| **n** | 29 | 29 | 29 | 29 | 29 | 29 | 29 | 29 | 29 | 29 | 7 | 29 | 29 |
| **Dung density** | 0.09454 | 0.07388 | 0.19282 | -0.25645 | 1 | 0.04022 | -0.02122 | 0.0213 | 0.19151 | 0.2899 | -0.23457 | 0.00068 | -0.04062 |
| **p** | 0.6257 | 0.7033 | 0.3163 | 0.1793 |  | 0.8359 | 0.913 | 0.9127 | 0.3196 | 0.1271 | 0.6126 | 0.9972 | 0.8343 |
| **n** | 29 | 29 | 29 | 29 | 29 | 29 | 29 | 29 | 29 | 29 | 7 | 29 | 29 |
| **Perennial basal gap size** | -0.73144 | -0.18271 | -0.57647 | 0.36772 | 0.04022 | 1 | 0.6492 | 0.44931 | 0.61013 | -0.28473 | -0.41927 | -0.31765 | 0.02329 |
| **p** | <.0001 | 0.3428 | 0.0011 | 0.0497 | 0.8359 |  | 0.0001 | 0.0145 | 0.0004 | 0.1344 | 0.3491 | 0.0931 | 0.9046 |
| **n** | 29 | 29 | 29 | 29 | 29 | 29 | 29 | 29 | 29 | 29 | 7 | 29 | 29 |
| **Perennial canopy gap size** | -0.56569 | -0.06664 | -0.47351 | 0.56662 | -0.02122 | 0.6492 | 1 | 0.08514 | 0.40333 | -0.26144 | -0.12627 | -0.34325 | 0.20945 |
| **p** | 0.0014 | 0.7313 | 0.0095 | 0.0014 | 0.913 | 0.0001 |  | 0.6606 | 0.03 | 0.1707 | 0.7873 | 0.0683 | 0.2755 |
| **n** | 29 | 29 | 29 | 29 | 29 | 29 | 29 | 29 | 29 | 29 | 7 | 29 | 29 |
| **BRTE cover** | -0.03072 | -0.37092 | -0.31555 | -0.34047 | 0.0213 | 0.44931 | 0.08514 | 1 | 0.09066 | -0.19125 | -0.35236 | 0.06218 | 0.02067 |
| **p** | 0.8743 | 0.0476 | 0.0785 | 0.0707 | 0.9127 | 0.0145 | 0.6606 |  | 0.64 | 0.3203 | 0.318 | 0.7486 | 0.9153 |
| **n** | 29 | 29 | 32 | 29 | 29 | 29 | 29 | 32 | 29 | 29 | 10 | 29 | 29 |
| **Annual forb cover** | -0.5135 | -0.07 | -0.36427 | 0.15225 | 0.19151 | 0.61013 | 0.40333 | 0.09066 | 1 | 0.00232 | -0.5 | -0.06715 | -0.0525 |
| **p** | 0.0044 | 0.7182 | 0.0521 | 0.4304 | 0.3196 | 0.0004 | 0.03 | 0.64 |  | 0.9905 | 0.2532 | 0.7293 | 0.7868 |
| **n** | 29 | 29 | 29 | 29 | 29 | 29 | 29 | 29 | 29 | 29 | 7 | 29 | 29 |
| **Perennial forb cover** | 0.22596 | -0.22806 | 0.35837 | -0.32411 | 0.2899 | -0.28473 | -0.26144 | -0.19125 | 0.00232 | 1 | -0.53546 | -0.05002 | -0.10216 |
| **p** | 0.2385 | 0.2341 | 0.0563 | 0.0863 | 0.1271 | 0.1344 | 0.1707 | 0.3203 | 0.9905 |  | 0.2155 | 0.7967 | 0.5979 |
| **n** | 29 | 29 | 29 | 29 | 29 | 29 | 29 | 29 | 29 | 29 | 7 | 29 | 29 |
| **PSSP cover** | 0.64134 | 0.57244 | 0.31735 | 0.46552 | -0.23457 | -0.41927 | -0.12627 | -0.35236 | -0.5 | -0.53546 | 1 | 0.41768 | 0.51761 |
| **p** | 0.1206 | 0.1793 | 0.3716 | 0.2925 | 0.6126 | 0.3491 | 0.7873 | 0.318 | 0.2532 | 0.2155 |  | 0.3511 | 0.2341 |
| **n** | 7 | 7 | 10 | 7 | 7 | 7 | 7 | 10 | 7 | 7 | 10 | 7 | 7 |
| **POSE cover** | 0.51155 | -0.05043 | -0.07657 | -0.24183 | 0.00068 | -0.31765 | -0.34325 | 0.06218 | -0.06715 | -0.05002 | 0.41768 | 1 | -0.25284 |
| **p** | 0.0046 | 0.795 | 0.693 | 0.2063 | 0.9972 | 0.0931 | 0.0683 | 0.7486 | 0.7293 | 0.7967 | 0.3511 |  | 0.1857 |
| **n** | 29 | 29 | 29 | 29 | 29 | 29 | 29 | 29 | 29 | 29 | 7 | 29 | 29 |
| **ELEL cover** | 0.07317 | 0.19967 | -0.19811 | 0.08483 | -0.04062 | 0.02329 | 0.20945 | 0.02067 | -0.0525 | -0.10216 | 0.51761 | -0.25284 | 1 |
| **p** | 0.706 | 0.2991 | 0.3029 | 0.6617 | 0.8343 | 0.9046 | 0.2755 | 0.9153 | 0.7868 | 0.5979 | 0.2341 | 0.1857 |  |
| **n** | 29 | 29 | 29 | 29 | 29 | 29 | 29 | 29 | 29 | 29 | 7 | 29 | 29 |

**b) Interspace microsites**

|  | **Perennial grass cover** | **ARTR density** | **rainfall** | **Bare ground cover** | **Cow pat density** | **Perennial basal gap size** | **Perennial canopy gap size** | **BRTE cover** | **Annual forb cover** | **Perennial forb cover** | **PSSP cover** | **POSE cover** | **ELEL cover** |
| --- | --- | --- | --- | --- | --- | --- | --- | --- | --- | --- | --- | --- | --- |
| **Perennial grass cover** | 1 | -0.24534 | 0.53948 | -0.591 | 0.09454 | -0.73144 | -0.56569 | -0.03072 | -0.5135 | 0.22596 | 0.54578 | 0.86069 | 0.06636 |
| **p** |  | 0.1996 | 0.0025 | 0.0007 | 0.6257 | <.0001 | 0.0014 | 0.8743 | 0.0044 | 0.2385 | 0.205 | <.0001 | 0.7323 |
| **n** | 29 | 29 | 29 | 29 | 29 | 29 | 29 | 29 | 29 | 29 | 7 | 29 | 29 |
| **ARTR density** | -0.24534 | 1 | -0.24538 | 0.1948 | 0.07388 | -0.18271 | -0.06664 | -0.37092 | -0.07 | -0.22806 | -0.34562 | -0.14745 | 0.0695 |
| **p** | 0.1996 |  | 0.1995 | 0.3112 | 0.7033 | 0.3428 | 0.7313 | 0.0476 | 0.7182 | 0.2341 | 0.4477 | 0.4453 | 0.7202 |
| **n** | 29 | 29 | 29 | 29 | 29 | 29 | 29 | 29 | 29 | 29 | 7 | 29 | 29 |
| **rainfall** | 0.53948 | -0.24538 | 1 | -0.3855 | 0.19282 | -0.57647 | -0.47351 | -0.31555 | -0.36427 | 0.35837 | -0.17396 | 0.53808 | -0.06758 |
| **p** | 0.0025 | 0.1995 |  | 0.0389 | 0.3163 | 0.0011 | 0.0095 | 0.0785 | 0.0521 | 0.0563 | 0.6308 | 0.0026 | 0.7276 |
| **n** | 29 | 29 | 32 | 29 | 29 | 29 | 29 | 32 | 29 | 29 | 10 | 29 | 29 |
| **Bare ground cover** | -0.59114 | 0.19483 | -0.38556 | 1 | -0.25645 | 0.36772 | 0.56662 | -0.34047 | 0.15225 | -0.32411 | 0.04278 | -0.66584 | 0.22152 |
| **p** | 0.0007 | 0.3112 | 0.0389 |  | 0.1793 | 0.0497 | 0.0014 | 0.0707 | 0.4304 | 0.0863 | 0.9274 | <.0001 | 0.2481 |
| **n** | 29 | 29 | 29 | 29 | 29 | 29 | 29 | 29 | 29 | 29 | 7 | 29 | 29 |
| **Dung density** | 0.09454 | 0.07388 | 0.19282 | -0.25645 | 1 | 0.04022 | -0.02122 | 0.0213 | 0.19151 | 0.2899 | 0.02507 | 0.31802 | -0.04151 |
| **p** | 0.6257 | 0.7033 | 0.3163 | 0.1793 |  | 0.8359 | 0.913 | 0.9127 | 0.3196 | 0.1271 | 0.9575 | 0.0927 | 0.8307 |
| **n** | 29 | 29 | 29 | 29 | 29 | 29 | 29 | 29 | 29 | 29 | 7 | 29 | 29 |
| **Perennial basal gap size** | -0.73144 | -0.18271 | -0.57647 | 0.36772 | 0.04022 | 1 | 0.6492 | 0.44931 | 0.61013 | -0.28473 | -0.28211 | -0.64507 | -0.0375 |
| **p** | <.0001 | 0.3428 | 0.0011 | 0.0497 | 0.8359 |  | 0.0001 | 0.0145 | 0.0004 | 0.1344 | 0.5399 | 0.0002 | 0.8469 |
| **n** | 29 | 29 | 29 | 29 | 29 | 29 | 29 | 29 | 29 | 29 | 7 | 29 | 29 |
| **Perennial canopy gap size** | -0.56569 | -0.06664 | -0.47351 | 0.56662 | -0.02122 | 0.6492 | 1 | 0.08514 | 0.40333 | -0.26144 | -0.15099 | -0.63097 | 0.42322 |
| **p** | 0.0014 | 0.7313 | 0.0095 | 0.0014 | 0.913 | 0.0001 |  | 0.6606 | 0.03 | 0.1707 | 0.7466 | 0.0002 | 0.0222 |
| **n** | 29 | 29 | 29 | 29 | 29 | 29 | 29 | 29 | 29 | 29 | 7 | 29 | 29 |
| **BRTE cover** | -0.03072 | -0.37092 | -0.31555 | -0.34047 | 0.0213 | 0.44931 | 0.08514 | 1 | 0.09066 | -0.19125 | 0.15807 | -0.09057 | -0.18793 |
| **p** | 0.8743 | 0.0476 | 0.0785 | 0.0707 | 0.9127 | 0.0145 | 0.6606 |  | 0.64 | 0.3203 | 0.6627 | 0.6403 | 0.3289 |
| **n** | 29 | 29 | 32 | 29 | 29 | 29 | 29 | 32 | 29 | 29 | 10 | 29 | 29 |
| **Annual forb cover** | -0.5135 | -0.07 | -0.36427 | 0.15225 | 0.19151 | 0.61013 | 0.40333 | 0.09066 | 1 | 0.00232 | -0.4067 | -0.33702 | -0.06862 |
| **p** | 0.0044 | 0.7182 | 0.0521 | 0.4304 | 0.3196 | 0.0004 | 0.03 | 0.64 |  | 0.9905 | 0.3652 | 0.0738 | 0.7236 |
| **n** | 29 | 29 | 29 | 29 | 29 | 29 | 29 | 29 | 29 | 29 | 7 | 29 | 29 |
| **Perennial forb cover** | 0.22596 | -0.22806 | 0.35837 | -0.32411 | 0.2899 | -0.28473 | -0.26144 | -0.19125 | 0.00232 | 1 | -0.35566 | 0.3341 | 0.19245 |
| **p** | 0.2385 | 0.2341 | 0.0563 | 0.0863 | 0.1271 | 0.1344 | 0.1707 | 0.3203 | 0.9905 |  | 0.4337 | 0.0765 | 0.3172 |
| **n** | 29 | 29 | 29 | 29 | 29 | 29 | 29 | 29 | 29 | 29 | 7 | 29 | 29 |
| **PSSP cover** | 0.54578 | -0.34562 | -0.17396 | 0.04278 | 0.02507 | -0.28211 | -0.15099 | 0.15807 | -0.4067 | -0.35566 | 1 | 0.41727 | 0.60811 |
| **p** | 0.205 | 0.4477 | 0.6308 | 0.9274 | 0.9575 | 0.5399 | 0.7466 | 0.6627 | 0.3652 | 0.4337 |  | 0.3516 | 0.1474 |
| **n** | 7 | 7 | 10 | 7 | 7 | 7 | 7 | 10 | 7 | 7 | 10 | 7 | 7 |
| **POSE cover** | 0.86069 | -0.14745 | 0.53808 | -0.66584 | 0.31802 | -0.64507 | -0.63097 | -0.09057 | -0.33702 | 0.3341 | 0.41727 | 1 | -0.11632 |
| **p** | <.0001 | 0.4453 | 0.0026 | <.0001 | 0.0927 | 0.0002 | 0.0002 | 0.6403 | 0.0738 | 0.0765 | 0.3516 |  | 0.5479 |
| **n** | 29 | 29 | 29 | 29 | 29 | 29 | 29 | 29 | 29 | 29 | 7 | 29 | 29 |
| **ELEL cover** | 0.06636 | 0.0695 | -0.06758 | 0.22152 | -0.04151 | -0.0375 | 0.42322 | -0.18793 | -0.06862 | 0.19245 | 0.60811 | -0.11632 | 1 |
| **p** | 0.7323 | 0.7202 | 0.7276 | 0.2481 | 0.8307 | 0.8469 | 0.0222 | 0.3289 | 0.7236 | 0.3172 | 0.1474 | 0.5479 |  |
| **n** | 29 | 29 | 29 | 29 | 29 | 29 | 29 | 29 | 29 | 29 | 7 | 29 | 29 |

**Variable definitions:**

*Perennial grass cover*: cover of perennial grasses, obtained through line-intercept sampling

*ARTR density*: density of *Artemisia tridentata* ssp *wyomingensis* (# / m^2^)

*Rainfall*: annual rainfall (inches)

*Bare ground cover:* % cover of bare ground, obtained through line-point-intercept sampling

*Cow pat density*: density of cow patties (dung) (# / m^2^)

*Perennial basal gap size*: length of gaps between bases of perennial vegetation (cm), obtained through gap-intercept sampling

*Perennial canopy gap size*: length of gaps between canopies of perennial vegetation (cm), obtained through gap-intercept sampling

*BRTE cover*: % cover of *Bromus tectorum*, obtained through line-point-intercept sampling

*Annual forb cover*: % cover of annual forbs, obtained through line-point-intercept sampling

*Perennial forb cover*: % cover of perennial forbs, obtained through line-point-intercept sampling

*PSSP cover*: % cover of *Pseudoroegneria spicata*, obtained by measuring cover in individual *Artemisia tridentata* canopies

*POSE cover*: % cover of *Poa secunda*, obtained by measuring cover in individual *Artemisia tridentata* canopies

*ELEL cover*: % cover of *Elymus elymoides*, obtained by measuring cover in individual *Artemisia tridentata* canopies
